# Simulated evolution assembles more realistic food webs with more functionally similar species than invasion

**DOI:** 10.1038/s41598-019-54443-0

**Published:** 2019-12-03

**Authors:** Tamara N. Romanuk, Amrei Binzer, Nicolas Loeuille, W. Mather A. Carscallen, Neo D. Martinez

**Affiliations:** 1 Department of Biology, Dalhousie University, Halifax, Canada; 2 Pacific Informatics and Computational Ecology Lab, Berkeley, CA USA; 3 Department of Physics, Chemistry and Biology, Linköping University, Linköping, Sweden; 4 Institute of Ecology and Environmental Sciences, Université Pierre et Marie Curie, Paris, France; 5 School of Informatics, Computing, and Engineering, Indiana University, Bloomington, IA United States

**Keywords:** Food webs, Ecological networks, Speciation

## Abstract

While natural communities are assembled by both ecological and evolutionary processes, ecological assembly processes have been studied much more and are rarely compared with evolutionary assembly processes. We address these disparities here by comparing community food webs assembled by simulating introductions of species from regional pools of species and from speciation events. Compared to introductions of trophically dissimilar species assumed to be more typical of invasions, introducing species trophically similar to native species assumed to be more typical of sympatric or parapatric speciation events caused fewer extinctions and assembled more empirically realistic networks by introducing more persistent species with higher trophic generality, vulnerability, and enduring similarity to native species. Such events also increased niche overlap and the persistence of both native and introduced species. Contrary to much competition theory, these findings suggest that evolutionary and other processes that more tightly pack ecological niches contribute more to ecosystem structure and function than previously thought.

## Introduction

Historically, prediction and management in ecology have been thought to be constrained mostly by demographic and ecological processes, with evolution playing a much more limited role or happening on much longer time scales^1^. In light of recent empirical studies, it is now widely recognized that evolution and ecology (and their interplay) affect the response of communities to environmental changes, and that the two processes may happen on similar timescales^2–4^. This is especially true when environmental changes occur at large scale or have high amplitude (e.g., current global changes), as selective pressures can then act efficiently to alter natural selection processes and/or species’ coevolution.

**Figure 1:**
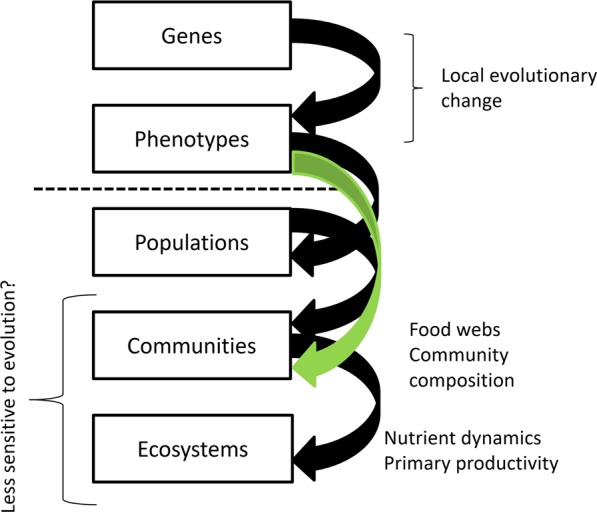
Evolution on ecological time scales influence populations and link contemporary evolution with community structure across levels of organization. Black arrows indicate more studied influences between adjacent organizational levels. For example, population genetics focuses on links above the dashed line between genes and phenotypes. The green arrow indicates the less studied links focused on here between evolutionary changes such as those in phenotype and more removed higher-level properties, such as community composition and food webs that may be less sensitive to evolutionary changes at lower levels.

Empirical examples suggesting the importance of evolution on ecological timescales are however usually focused on either one species responding to changes in the abiotic environment^5,6^ or on one interaction^7,8^. These studies strongly suggest evolution alters the fate of the studied population, which links the individual gene/organism level with population structure but leaves unanswered how such effects might propagate to higher levels of organization such as communities or ecosystems (Fig. 1). For example, such propagation may occur in more complex trophic modules when local evolutionary adaptation modifies community structure and ecosystem functioning by altering competitive interactions and enabling predators to more strongly reduce prey abundance^9^ and when different intraspecific genetic variants of key species alter the structure and functioning of an ecosystem^10,11^. Analyses of complex food webs suggest that strong selection on fish’s life history due to fishing may cause evolutionary processes to decrease catch and destabilize the functioning of fishery ecosystems by selecting for small body size and early maturation of fished populations^12^. These observations link evolutionary processes to communities and ecosystem function^13^ (Fig. 1).

In general, such linkage between organizational levels may critically depend on the degree to which mutation and/or speciation introduces organisms into ecological systems that are functionally distinct from organisms that migrate into the system. Migration due to changes in the environment e.g., climate change^14^, stochastic dispersal processes, or human facilitation can rescue populations that might otherwise go extinct or add new functional groups to the community^15,16^. Both of these effects can alter other species’ densities or interactions. Evolution can also cause these effects by altering interactions, e.g. evolution of diet^17,18^, while also altering intraspecific phenotypic and genetic diversity^10,19^. We focus here on forms of evolution that may alter a population’s phenotype such as sympatric and parapatric speciation within local communities or ecosystems, and we focus on these local systems in terms of their trophic interactions, food webs, and the degree to which evolution and migration may alter the structure and robustness of food webs.

The role of evolution in community assembly, such as new species being introduced via local speciation, has been explored more recently and less extensively than the role of invasions, i.e., introductions from other communities within a region^20–22^. Community assembly processes may vary from being almost completely dominated by introductions from nearby source pools over the short time scales to being largely dominated by speciation after long periods of time in isolated environments very far from source pools^23^. Several^16,24–28^ but not all^29^ eco-evolutionary theories of community assembly describe dynamics where new phenotypes are introduced and potentially establish within the community and alter the community’s network structure and dynamics depending on selective pressures formalized as ordinary differential equations. One type of community evolution model relies on a large number of biologically undefined phenotypic traits free of trade-offs^24,25,30^. Clearer interpretations emerge from another type of model that relies on one or a few biologically defined traits such as body size^28,31–35^ which can produce persistent and empirically realistic networks^31,34^ and elucidate how coevolution of species may affect community stability^34,36,37^. Fundamental similarities among these trait-based studies of community coevolution include the introduction of new phenotypes into a community and subsequent selection based on the population dynamics that emerge from ecological interactions structured according to traits of each phenotype or “species” within the community.

While the introductions and dynamics of species in evolutionary and ecological community assembly models are similar, they differ with respect to the variability of species that are introduced^15,27^. Introductions in community evolution models typically mimic speciation or mutation events derived from ancestral species within the community by slightly modifying that ancestor’s traits^31,34,38^. This simulates phylogenetic conservation of niches^39^ by typically introducing species whose function is similar to at least one species within the community in terms of life history and interactions. New functional groups may arise^31^, but most likely by progressive selection events operating on relatively similar species. By contrast, community assembly models typically introduce species whose traits such as preferences for prey and vulnerabilities to predators are relatively uncorrelated with traits of species already within the network. Such ecological assembly often adds more variability to the community including new functional groups more immediately than does evolutionary assembly. In both cases, phenotypes more or less similar to those already in the community are introduced and then subjected to selection determined by species interactions structured according correspondence of species’ traits.

We use this framework of functional similarities and differences^15,40^ to focus on how similarity of introduced species to those residing in the food web affects the robustness and structure of the assembled network^41^. We account for evolutionary processes responsible for phenotypic variation by assuming that niche conservatism^39^ causes “speciation” events to introduce species whose niches are more similar to at least one of the species of the network while “invasion” events introduce species whose niches are typically more different from species within the network. We focus on two main questions: (1) Are food webs assembled through invasions more or less persistent compared to food webs assembled by speciation events? (2) Is the structure of “invasion” networks more or less similar than “speciation” networks to empirically observed networks? Based on competitive exclusion principles that assert species are less likely to persist the more they share niches with resident species^15,40–42^, one expects that species introduced via speciation events cause more extinctions and are therefore less persistent than introductions of invaders who share less of their niche space with resident species. However, invasions may cause a larger disturbance than speciation events due to the greater functional difference between invaders and resident species. Therefore, one may expect that “invasion” webs should be less persistent. Our results support this latter prediction and indicate that the mechanism can be more finely understood by accounting for distribution of vulnerability and generality of the newly introduced type. Our results also show that “speciation” networks more closely resemble empirical food webs. These findings have important implications for invasion ecology and co-evolution within complex communities^22^.

**Figure 2:**
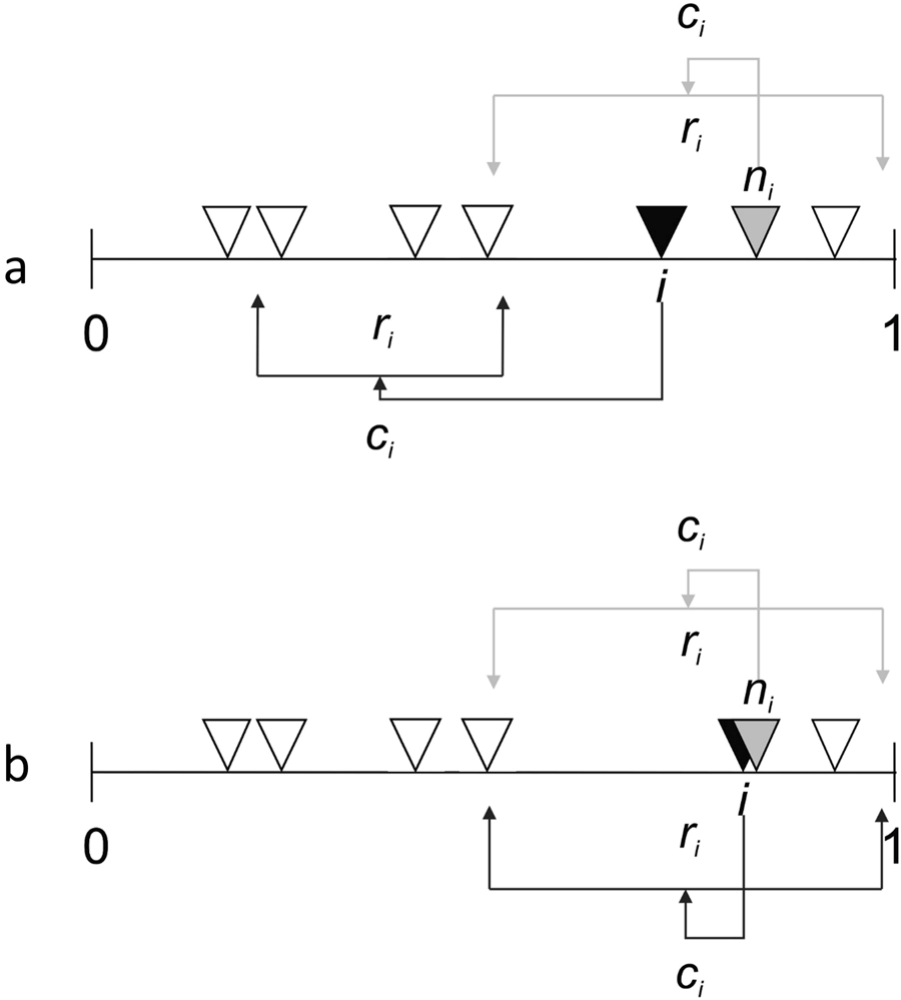
We use the stochastic “niche model”^43^ with inputs of species richness (*S*) and directed connectance (*C* = # of links/*S*^2^)^45^ to generate food webs. Each *i*^th^ of *S* species shown as a triangle is assigned a randomly drawn ‘niche value’ (*n*_i_) from the interval (1,0). Each *i*^th^ species is then constrained to consume all prey species within a range of beta-distributed values (*r*_i_) whose mean is *C* and whose randomly chosen center (*c*_i_) is less than the consumer’s niche value. If an introduced node shown as a black triangle differs greatly from other nodes (e.g., gray triangle) in the network, we assume this node should be considered as (**a**) an ‘invasion’ event whereas (**b**) if an introduced node is largely similar to another node already in the network the new node represents a ‘speciation’ event.

We conducted our simulations in three steps. First, we generated three sets of realistically structured networks^43^ with 35 species with low, medium, and high levels of connectance (fraction of all possible links realized) along with three corresponding sets of species whose traits such as trophic niche width enabled them to be introduced and trophically linked to species in each web with minimal methodologically enforced changes to their connectance (Fig. 2). Allowing introductions of species from different connectance classes would systematically change connectance levels during introduction sequences. We avoid such changes to better represent an ecological realistic range of connectance, which may reflect different habitats or regional biotas with systematically different biotas. For example, soil food webs appear to have unusually high connectance^44^ and contain species unable to survive in lakes with lesser connectance^45^ or in above ground communities found to have much lower connectance^46^. We avoid distinguishing connectance classes further because results at each connectance level are qualitatively identical and quantitatively very similar (Fig. S1). Our second step used allometric trophic network models^47–49^ to simulate species’ population dynamics within each network for 2000 time steps, which generated dynamically persistent food webs with 30 species that are remarkably similar to empirical food webs^50^. Our third step uniformly randomly chose 30 species from a set of species created in the first step and sequentially introduced one from this set every 200 time steps into each of 100 persistent webs from each of the three levels of connectance. The simulations were then stopped 2000 time steps after the last introduction. Extinctions were measured as the number of species that went below our threshold of 10^−30^ during the simulations.

Given the centrality of niche overlap to eco-evolutionary theories of community assembly^41,51,52^, we calculated the niche similarity of the introduced species upon introduction to its most trophically similar species already in the web^45^. This “maximum similarity” of an introduced species is the number of predators and prey shared in common divided by the pair’s total number of predators and prey^45,53,54^. We assume introducing trophically dissimilar species represents longer range dispersal events of species arriving to the community from a regional species pool while introducing trophically similar species represent sympatric or parapatric speciation events hereafter referred to as ‘speciation’^15^. Overall characterization of each introduction sequence as either invasion or speciation dominated was based on the average maximum similarity of all 30 introduction events for each web, hereafter called “mean maximum similarity.” See Methods for additional details.

## Results

**Figure 3:**
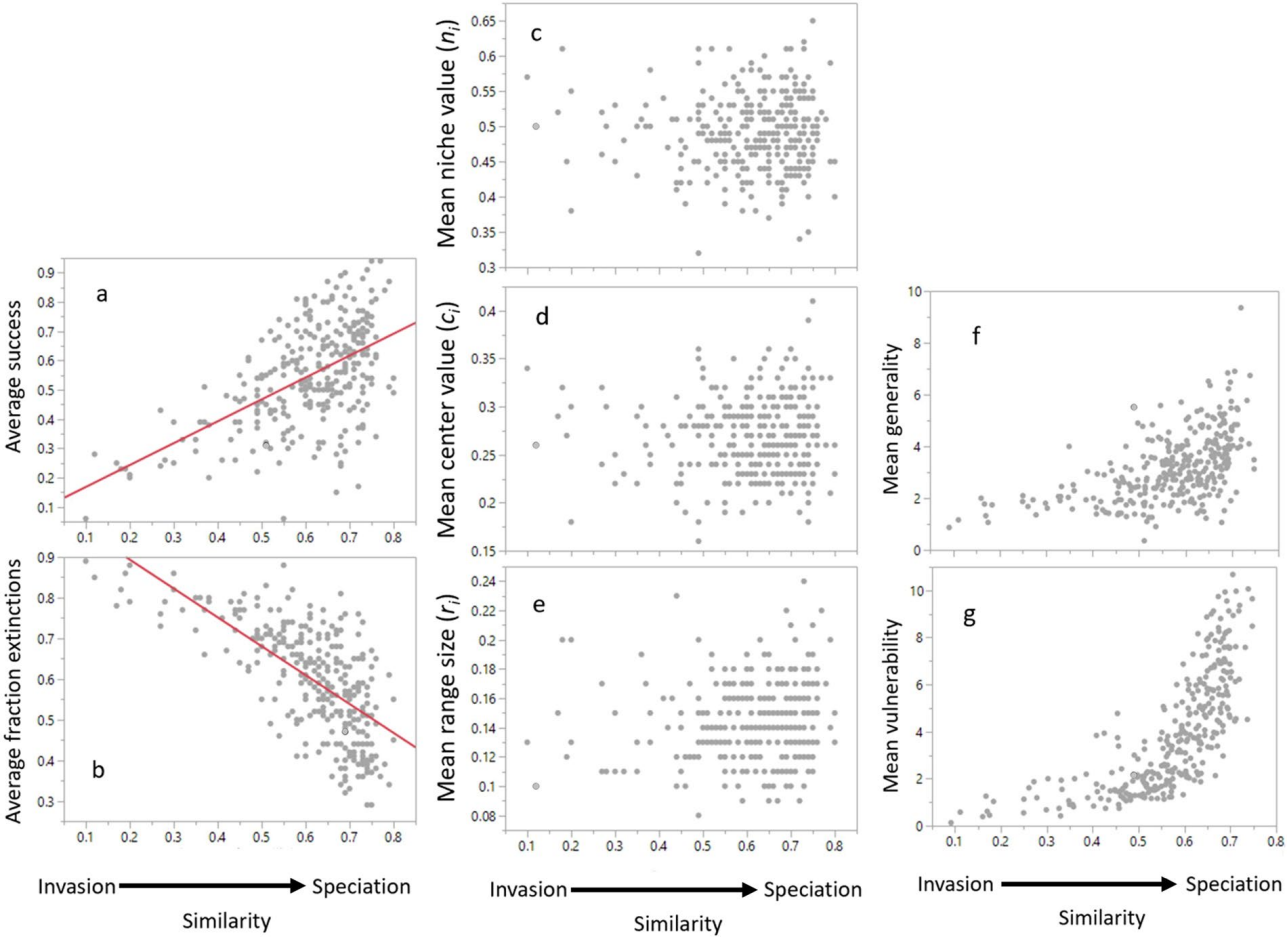
Relationship between niche overlap and properties of introduced species. Overlap is measured by the mean maximum similarity (mean of the fractions of trophic links shared between each of the 30 introduced species and the species most similar to them already in the food web). Mean maximum similarity on the x-axis describes a gradient from invasion dominated to speciation dominated webs. Data for all connectance classes are presented and each data point is an average of the properties across all 30 introductions. (**a**) Species with higher similarity (speciation) are more successful and (**b**) cause fewer extinctions than assembly with species from a regional species pool (invasions). No strong directional trends from more dissimilar to more similar species appear in the mean niche-model properties of the introduced species’ (**c**) niche value, (**d**) size of feeding range, and (**e**) location (increased location correlated with increased trophic level) of the feeding range. Mean number of species within the web that eat (vulnerability, **g**) and is eaten by (generality, **f**) the introduced species increases with the mean similarity of those species to species already in the web indicating that species with higher similarity feed on more species and are fed on by more species which may diffuse interaction strengths among species.

Compared to introductions of invaders, introductions of speciated species were typically both more successful in that they much more likely persisted through to the end of the simulations and also less disruptive in that their introduction caused fewer extinctions (Fig. 3). This success is shown by mean maximum similarity being significantly and positively correlated with the proportion of introduction events that were successful (r^2^ = 0.3, p < 0.0001; Fig. 3a). In some cases, more than 90% of the introduced species in speciation-dominated webs were successful. The reduced disruption is demonstrated by the significant decrease (r^2^ = 0.45, p < 0.001; Fig. 3b) in the average fraction of species extirpated in webs during each introduction sequence as mean maximum similarity increases. Overall, webs assembled by invasion lost more species and the introductions themselves were much less successful than in webs assembled by speciation.

Considering both the properties of the introduced species and the properties of the webs themselves^55^, such as their connectance, helps inform the interpretation of these results (Fig. 3). Our analyses showed that fundamental properties^56^ of the introduced species differed little between invasion-dominated and speciation-dominated webs (Fig. 3c–e). This is demonstrated by the lack of significant correlations between mean maximum similarity of the introduced species and the three niche model parameters of the invader that dictate the introduced species’ location (*n*_*i*_; r^2^ < 0.001, p = 0.943, Fig. 3c) as well as the size (*r*_*i*_; r^2^ = 0.011, p = 0.068, Fig. 3e), and location (*c*_*i*_; r^2^ = 0.0008, p = 0.615, Fig. 3d) of its feeding range along the niche dimension (Fig. 2). The lack of such correlations suggests that fundamental properties such as where introduced species are in community niche space, the breadth and location of their feeding range within this space explains little of the variability while how these traits relate to traits of species already in the web explains much more. This pattern is supported by clear patterns in the realized niche properties of introduced species along the similarity gradient (Fig. 3). The generality (r^2^ = 0.287, p = 0.001; Fig. 3f) and vulnerability (r^2^ = 0.52, p < 0.001; Fig. 3g) of the introduced species as it entered the web were strongly and positively correlated with mean maximum similarity. Interestingly, these correlations were similar for both initial and final realized niche properties for the introduced species. For example, final generality was also strongly and positively correlated (r^2^ = 0.21, p < 0.001) with the mean maximum similarity of the introduced species. Part of this pattern would seem to emerge from more general and vulnerable species having more interactions which would overlap with species already in the web and therefore correlate well with mean maximum similarity. Conversely, species with low generality and vulnerability would have less potential for overlap and therefore low mean maximum similarity. However, more trophic interactions can also increase differences between introduced and resident species which can counteract correlations between increased numbers of interactions and increased mean maximum similarity. Whichever is the cause of the strong correlations between the number of interactions of introduced species and their maximum similarity to resident species, our results suggest that sharing large numbers of interactions with resident species facilitates rather than inhibits their persistence in the networks and reduces the number of extinctions caused by their persistence. On the other hand, fewer and more unique sets of interactions appears to inhibit introduced species from persisting and increase the number of extinctions caused by their introduction. Additionally, possessing relatively unique sets of interactions appears highly unlikely due to chance alone and instead appears associated with the structure of the resident community rather than introduced species possessing unusual fundamental niche properties. This is illustrated by the many fewer introductions at the invaders’ side of the similarity axis than at the speciated species’ side (Fig. 3a,b) despite the absence of significant trends in fundamental niche values along the same axis (Fig. 3c–e).

**Figure 4:**
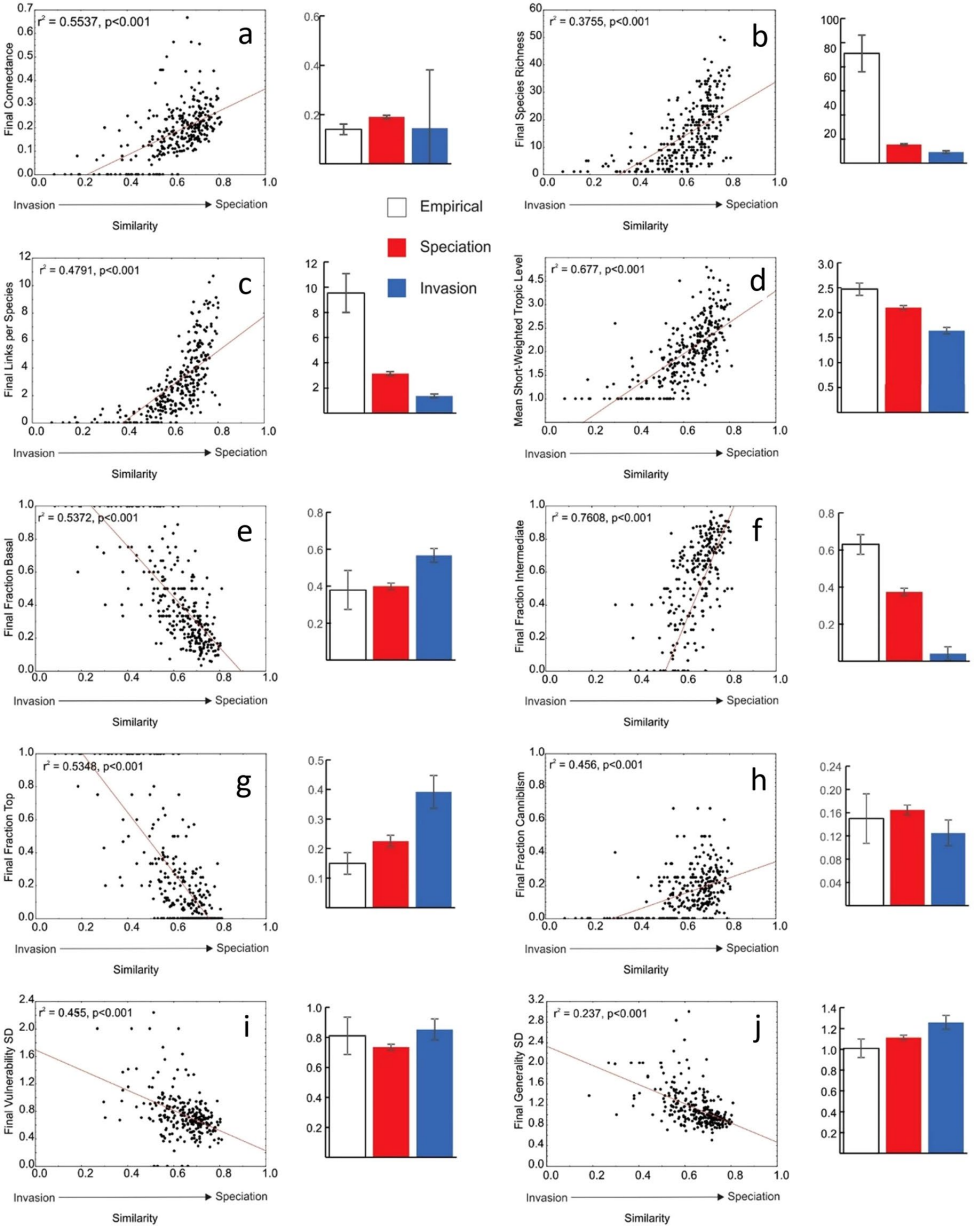
Food web properties of invasion dominated versus speciation dominated webs at the end of the simulations compared with those in empirical webs from the literature. Error bars indicated 95% confidence intervals. Significant differences occur between properties whose error bars do not overlap. Here, we consider invasion-dominated webs to have mean maximum similarity of introduced species <0.5 and that of speciation-dominated webs to be >0.5. This choice balances fewer networks within a larger range (0.1–0.5) of similarity with more networks within a smaller range (0.5–0.8) Still, qualitatively similar conclusions are reached using the average (0.61) or median (0.64) of all networks’ mean maximum similarities as a boundary between invasion- and speciation-dominated food webs.

The differences in the types of introduced species that were able to persist and the impacts of their introductions along the assembly gradient from invasion to speciation also created differences in final food web structure along this gradient (Fig. 4). This is shown by several of the most fundamental and ecologically informative among many metrics of food web structure^43,57^ measured at the end of the simulations including connectance (*C*, Fig. 4b), species richness (*S*, Fig. 4b), and mean trophic level (SWTL; Fig. 4d). These results show that, by the end of the simulations, speciation-dominated webs have more species, higher connectance and a higher average trophic level.

A key consideration is whether community assembly due to invasion or speciation differs in terms of how close the assembled communities resemble empirical communities and each other in terms of their food web structure. To address this consideration, we used a set of published values for food web structure to assess differences in ten food web properties among 19 empirical webs^57^ as well as for webs assembled along the speciation and invasion spectrum (Fig. 4). For most food web properties, empirical webs differed from both the speciation- and invasion-dominated webs. However, in general, the food web properties for speciation-dominated webs were more similar to the empirical webs than the properties for invasion-dominated webs. Of the 10 properties we assessed, 5 including *S* (Fig. 4b), links per species (Fig. 4c), mean short-weighted trophic level (Fig. 4d), and the fractions of intermediate (Fig. 4f) and top (Fig. 4g) show significant differences among invasion- and speciation-dominated and empirical food webs. Speciation-dominated webs are closer to empirical webs for all 4 of these properties. Another 2 properties, fraction of basal species (Fig. 4e) and the standard deviation of generality (Fig. 4j), were similar for empirical and speciation dominated webs and also significantly lower than that for invasion-dominated webs. The relative match of the remaining three properties including connectance (Fig. 4a), cannibalism (Fig. 4h), and the SD of vulnerability (Fig. 4l) to empirical webs was less conclusive. Still, the means for the ten properties indicate that speciated networks are more similar to empirical networks for all but two properties; connectance (Fig. 4a) and the standard deviation of vulnerability (Fig. 4l).

Overall, properties at the end of the assembly sequence for invasion-dominated and speciation-dominated webs (Fig. 4) indicate that speciation-dominated webs are more similar to empirical webs by having more species, more links per species, higher mean trophic levels, a lower proportions of basal and top species, more intermediate species, more cannibalism, and lower SD for generality than invasion-dominated webs. This shows that speciation-dominated webs are more complex and reticulate than invasion-dominated webs.

## Discussion

Overall, a number of differences were observed in assembly dynamics and final food web structure along the mean maximum similarity (invasion to speciation) gradient. In particular, webs dominated by speciation events had a higher proportion of successful introductions and fewer extinctions, which resulted in webs that tended to have more species, higher connectance and higher trophic levels. Compared to the low generality and vulnerability of invading species, species introduced by speciation had higher generality and vulnerability which suggests that speciation introduces more interactions than invasions. Despite the possibility for increased interaction density to increase differences among species, such density creates more trophic overlap and helps explain the high trophic similarity of speciated species of speciation dominated webs. The structure of speciation-dominated webs more closely resembled those of a set of empirical food webs. This suggests that evolutionary dynamics may play a larger role in the structure and function of food webs than previously thought but appear opposite to expectations based on competitive exclusion^15,40,41,58^. Such expectations include niche partitioning where competition between species that share niches cause extinctions while species that avoided such overlap by partitioning niches would allow more species to persist^15,40,41,58^. However, these expectations may be due to incorrect interpretation of Lotka-Volterra models that simplify indirect effects between consumers sharing resources by translating them as direct interactions between consumers^59^. We avoid such problems here by “working with models that better encapsulate the mechanisms of interactions among multiple species” as suggested by others^59^.

Absent potentially overstated difficulties of sharing niches^41,42^, our findings may result from niches that enable species to persist being already occupied in our native webs which restricts successful introductions to be those that join residents of already occupied niches. Conversely, introduced species with different niches than residents are more likely to occupy niches that are dynamically unable to sustain species. Others have suggested that competition can cause convergent evolution to lead to sympatric speciation^40^ for which there is substantial field evidence^40,58^. Another possibility is that the relatively small non- or less overlapping parts of similar species’ niches provide sufficient resources for their sustenance as in pollination networks^60^. High generality could help maintain this sustenance by enabling species to shift their feeding towards less shared resource species whose identity may vary during dramatic changes that may accompany repeated introductions. This key role of small parts of generalists’ niches with a few strong links and many weak links is consistent with the stabilizing effect of low mean interaction strength of species with many interactions^61^. While future studies need to better explore these and other potential explanations, our findings add to other findings indicating that sharing much of one’s niche with other species, as one may expect in cases of sympatric and parapatric speciation, does not appear to strongly prevent species from coexisting and dynamically persisting^15,40,58,62^.

Invasions in our simulations dramatically affect the food web in several ways similar to invasions in nature by causing secondary extinctions that severely reduce the diversity of the native community^63,64^ and by greatly simplifying and reducing the number of trophic levels in the community’s overall structure. A prominent example is the invasion by Burmese pythons of the Florida Everglades (USA) which substantially altered the abundance distribution of its prey^65^. Another is the invasion of Australia by cane toads whose toxicity reduced the abundances and reorganised the communities of the toads’ predators^66^. These observations anecdotally suggest that invasions by species substantially different than native species can greatly alter the local network structure and diversity in nature qualitatively similar to those in the present study.

Beyond such effects on communities, invasions often fail by not persisting within the system as seen where invading species remain at low populations for extended periods^67^ or need several attempts to become an effective invader^68^. Our results combined with the frequent implicit assumption that phylogenetic distance is strongly related to ecological similarity in general^69^ and to network similarity in particular^70,71^ suggests that invasion is easier for species that are phylogenetically close to one of the local species in agreement with recent data^62,72^ used in invasive species prevention schemes^73^.

Our results showing that networks assembled through speciation events are more similar to empirical networks agrees with other community evolution models that showed that evolutionary dynamics allow realistic network structures to emerge^30,31,34,74,75^. However, our evolutionary rules differ from these former models by relying on interaction similarity of phenotypes that emerge from evolutionary and ecological processes such as speciation and long range dispersal rather than relying on more explicitly modeled dispersal^16^ or evolutionary dynamics of traits such as body size^31,75^, foraging traits^34^ or competition based on interaction distributions^74^. Still, as in our study, these evolutionary events introduce new species whose niches substantially overlap with established species but whose traits such as body size, predators, and prey may slightly differ. While the rules generating new species differ, the basic co-evolutionary structure of these studies share the fundamental components of introduced trait variation and selection based on interaction with all species in the community. The bioenergetic basis of these studies ensure that such variation is retained over multiple generations and, as such, is effectively inherited. Disparate approaches to studying these fundamental components of evolutionary dynamics all lead to at least somewhat empirically realistic structures. This consistency suggests that a key aspect this process, i.e., a large degree of niche similarity between new and native species or morphotypes, is important to assembling realistic networks.

When the network is assembled by speciation events, community robustness increases as evidenced by fewer secondary extinctions^76^ which leads to more complex trophic structures. While this stabilizing effect of evolution may not be expected generally in diverse communities, it appears more likely in trophic networks^77^. Evolution in food webs leading to stable and complex structures has been observed in many different models, relying on very different sets of rules^30,31,36,78^. Introducing species into bipartite networks of plants and pollinators found more conventionally expected results where increased niche overlap among pollinators led to less persistence of native species^79^. However, this only occurred when there was an extraordinary difference in the invader’s niche beyond niche overlap; the cost-free ability to feed twice the rate of native pollinators. This ability caused the invaders to extirpate all natives whose niches were a subset of the invader’s niche and greatly reduce the abundance of other natives whose niches only partly overlapped the invader’s niche who survived by shifting their feeding to plants not consumed by the invader. Higher trophic levels (e.g., carnivores) may reduce such dramatic effects by preventing competition within lower levels from extirpating species^80,81^ and weakening the strong interactions that destabilize complex food webs^77,82,83^. Our analysis advances this latter idea by suggesting that such balance more specifically concerns maintaining high levels of vulnerability and generality between new species and their progenitors.

Our finding regarding “speciation” and “invasion” networks may be tested against natural systems greatly contrasting in their openness to migration. For very closed networks (e.g., very isolated islands with no humans that introduce invasive species), species invasion may be quite rare, so that the network may mostly represent the effects of species local adaptation or coevolution. Our “speciation” results may best match such closed networks in e.g., showing largely stable and complex structures. On the other hand, we expect very open networks (e.g., a continental ecosystem at the crossroad of many migratory paths or close to many human populations) may have much more frequent invasions causing extinction cascades as well as having a lower complexity with evolution playing a secondary role compared to invasions.

We emphasize here our qualitive results largely because they are relatively insensitive to the uncertain boundary between similarity extremes that may distinguish invasion from speciation (Fig. 4). However, we also note that feedbacks between these two extremes may strongly influence evolution by changing the community structure and thereby altering how coevolution propagates through the network^84^. New predators may alter defense strategies among native prey as has been seen where mussels evolved thicker shells in response to the invasion by the Asian crab^85^. Similarly, predators may evolve in response to an invasive prey as predators have to invasive cane toads^86^. Our use of average similarity of 30 different invaders and the spectrum from invasion- to speciation-dominated webs helps inform such interactions between invasion and speciation by suggesting how webs generated by more interactions between these two types of species introductions are intermediate between webs mostly generated by one type of introduction.

This interplay of invasion and evolutionary processes may help predict future states of ecological networks e.g., whether communities become closed or otherwise resistant to future invasions. Simulated invasions often do not cause such community closure both due to cyclic behaviours^87^ and because drawing rates at random allow for infinite combinations. On the other hand, evolutionary dynamics may be trapped in local maxima of fitness which prevents introduced species or morphotypes from differing greatly from resident populations^40^. This greatly restricts the sampling of the possible parameter space by evolutionary processes. For these reasons, it has been suggested that allowing for a mix of invasions and evolution may be key to understanding community closure^22^. Our approach may be easily adapted to tackle this type of question by focusing how assembly changes over time rather than more simply comparing average outcomes between beginning and end states.

More broadly, our results add to other findings suggesting that, whether assembled by ecological or evolutionary mechanisms, surprisingly large numbers of species are able to coexist despite large amounts of niche overlap^15,40,58^, in our case both in terms of resource and consumer species, the latter of which has received little previous attention. For example, Morlon *et al*.^52^ found that species in a region’s 50 lake communities share prey much more often than expected if community composition resulted from randomly sampling all species from all 50 lakes within the region. Such findings taken together suggest that there are strong ecological and evolutionary mechanisms forcing species to fit within a relatively restricted architecture of trophic niches as described by theory such as that formalized by the trophic niche model (Fig. 2), which has much higher niche overlap than more randomly structure niche architectures^43,88–91^. Further research into these and other mechanisms that increase interspecific functional similarity and its consequences may greatly elucidate processes and patterns within complex natural ecosystems.

## Methods

The behavior of many if most networks from protein-protein interaction networks to large-scale power grids critically depend on their structure and food webs are no exception^92^. To provide such structure, we use the niche model^43^ (Fig. 2) which is based on two basic ecological processes. The first involves the bioenergetic mechanisms responsible for feeding inefficiencies that create a trophic hierarchy above the autotrophic source of all heterotrophic food. The second involves phenotypic mechanisms such as gape size that restrict carnivores to consuming a contiguous range of body sizes or leaf composition that may restrict herbivores to phylogenetically related plants such as grasses. These mechanisms are formalized by hierarchically structuring feeding interactions according to trophic levels with species at higher levels feeding on species mostly below them within contiguous ranges of the hierarchy. Based on only two input parameters and free from any tunable parameters, the niche model closely predicts food-web structure including many empirically observed patterns observed in highly resolved food webs^43,88,90^ including those from a half billion years ago^89^. These patterns range from food chain length and the distribution of species among trophic levels to the variation between trophic specialization and generality. As such, the niche model provides the most realistically structured networks available for ours and other similar^34^ studies. The niche model does this by randomly assigning each of *S* species (*i*) in a food web a niche value (${n}_{i}$) which places the species in a uniformly random position within a one-dimensional ‘community niche space’ from 0 to 1 (0 ≤ ${n}_{i}$ ≤ 1). Species *i* eats all species whose niche value falls within a feeding range (${r}_{i}$) whose center (${c}_{i}$) is a uniformly random number between ${r}_{i}/2$ and min (${n}_{i}$, $1-{r}_{i}/2$), which ensures that $\,{c}_{i}\le {n}_{i}$, and that ${r}_{i}$ fits entirely within the community niche space. In other words, species *i* eats species *j* when *n*_*j*_ is between *c*_*i*_ ± ${r}_{i}/2$. These constraints on ${c}_{i}$ biases *i*’s diet towards species with *n*_*j*_ < *n*_*i*_. Looping and cannibalism occur when *i*’s feeding range includes species with *n*_*j*_ ≥ ${n}_{i}$. Since $\,{r}_{i}={x}_{i}{n}_{i}$ where 0 ≤ ${x}_{i}$ ≤ 1 is a random variable with a beta-distributed probability, species with high *n*_*i*_ tend to eat more species^43,88^.

Population dynamics follow Yodzis and Innes’^93^ non-linear consumer-resource model with updated allometric coefficients^47,49^ and extended to include many species^56,94^ and a producer-nutrient model for basal species dynamics^48,80^. Rates of production, metabolism and maximum per capita consumption scale with species body mass according to a 3/4 power law^95^. A species’ body mass increases with its trophic position with a mean of 10^2^ and a standard deviation of 10^1^. Species with no species within their *r*_*i*_ are assigned to be autotrophic basal species that consume two primary limiting nutrients with a fixed rate of input. Consumption rates depend on abundances of the consumers and resources according to a saturating functional response. Our model belongs to a family of “allometric trophic network” models noted for their successful predictions of the effects of experimental species removals in the field^96^ and simulations of seasonal dynamics of a complex food web within a temperate lake^49^.

More precisely, we model changes in species biomass densities (${B}_{i})\,$ over time as:

1 $$\frac{d{B}_{i}(t)}{dt}={G}_{i}(B)-{x}_{i}{B}_{i}(t)+\mathop{\sum }\limits_{j}^{n}({x}_{i}{y}_{ij}{F}_{ij}(B){B}_{i}(t)-\frac{{x}_{j}{y}_{ji}{F}_{ji}(B){B}_{j}(t)}{{e}_{ji}})$$

where ${G}_{i}(B)$ is non-zero only for basal species whose growth rate is^47,80^:

2 $${G}_{i}(N)=MIN(\frac{{N}_{1}}{{K}_{1i}+{N}_{1}},\frac{{N}_{2}}{{K}_{2i}+{N}_{2}})$$

where ${N}_{1}$ and ${N}_{2}$ are the concentration of two limiting nutrients, and ${K}_{1i}$ and ${K}_{i2}\,$ are species *i*’s half saturation densities for nutrient 1 and 2, respectively. *N*_*l*_ varies according to:

3 $$\frac{d{N}_{l}(t)}{dt}=D({S}_{l}-{N}_{l})-\mathop{\sum }\limits_{i=1}^{n}\,{c}_{li}{r}_{i}G(N){B}_{i}$$

where chemostat-like nutrients flow into the nutrient pool at a rate of *D* relative to the growth rate of a chosen producer species times a nutrient supply concentration of *S*_*l*_ and out of the pool at the same rate of *D* times *N*_*l*_. Uptake from available nutrients is determined by the right-most term in Eq. 3 where *c*_*li*_ is the concentration of nutrient *l* in the biomass of species *i*.

The second term in Eq. 1 is the metabolic loss where ${x}_{i}$ is *i*’s mass-specific metabolic rate. The third and fourth terms are the gains from resource consumption and loss to consumption, respectively, where ${y}_{ij}$ is consumer *i*’s maximum consumption rate of species *j*, relative to consumer *i*’s metabolic rate. ${e}_{ij}$ is *i*’s assimilation efficiency when consuming species *j*. The functional response, ${F}_{ij}(B),$ describes the realised fraction of *i*’s maximum rate of consumption achieved when consuming species *j*. Since Holling^97^ established the modeling framework for the functional response, several variations of the response have been used^94,98,99^. Similar to Romanuk *et al*.^56^, we use a modified “type II.2” functional response, which is close to a type II response but provides much of the stability of a type III response^50^. This response models consumption of resource *j* by consumer *i* as

4 $${F}_{ij}(B)=\frac{{B}_{j}^{h}}{{\sum }_{k=resources}\,{B}_{k}^{h}+{B}_{0}^{h}}$$

Where ${B}_{0}$ is the half saturation density and $h\,$ is the Hill exponent where $\,h=1.2$ which is a well-studied intermediate^80,94,100^ between Holling type II (*h* = 1) and III (*h* = 2) responses. The amount that each resource *j* loses to consumer *i* is equal to the resource’s density, $\,{B}_{j}$, divided by the sum of all the densities of the consumer’s resources times the consumer’s rate of consumption. This allows a species to consume at its maximum rate even if only one of its prey has a high biomass^101^. The last term of Eq. 1 describes the loss of species *i*’s biomass to consumer *j* which is equal to consumer’s consumption rate divided by *e*_*ji*_, the efficiency of that consumption.

We created three sets of food webs by parameterizing the niche model with 35 species (*S*) and connectance (*C* = *L*/*S* ^2^ where *L* is the networks’ total number of feeding links) values of 0.05, 0.10, or 0.20 for each set. Persistent webs where generated by initializing each species with randomly chosen biomasses between 10^−2^ and 10^−3^ and extirpating species whose abundance decreases below 10^−30^. The first 110 webs within the set parameterized within each connectance value that maintained a species richness of 30 after 2000 time steps were retained for the additional simulations steps. Within each connectance class, the first 100 webs were chosen to be subjected to introductions and the 10 remaining webs served as a source for introductions. We subjected each of the 100 webs to a sequence of 30 introductions by 30 different species randomly chosen from the 300 species within the 10 source webs. The position of each introduced species within the web emerged from following the niche model’s rules (Fig. 2) for how the introduced species’ traits (*n*_*i*_*, r*_*i*_*, and c*_*i*_) relate to the traits of species residing in the web. Body masses of species and other parameters are chosen based on the scaling of body size with trophic level^80,96^ where each consumer-resource body size ratio is chosen from a normal distribution with mean of 10^2^ (SD = 10^1^). Following ref. ^93^, we assigned *B*_0_ = 0.5, *y*_*ij*_ = 8, *e*_*ij*_ = 0.45 for consumption of basal species, and *e*_*ij*_ = 0.85 for consumption of non-basal species. Following ref. ^47^, we assigned initial *S*_*l*_ = *N*_1_ = *N*_2_ = 1, *K*_1*i*_ = *K*_2*i*_ = 0.15, *D* = 0.25, *c*_1*i*_ = 1, and *c*_2*i*_ = 2.

The first of the 30-species sequence was introduced after the initial 2000 time steps required to generate persistent webs at *t* = 2001 with an initial biomass uniformly randomly chosen between 1 × 10^−7^ and 1 × 10^−9^. Thereafter, one more species from the sequence was similarly introduced every 200 time steps. These 200 steps allow the vast majority of introductions to reach steady state before the next introduction^96^. The simulations continued another 2000 times after the last introduction so that simulations lasted 10,000 time steps (until *t* = 10,000) while maintaining the extinction threshold at 10^−30^. We repeated this procedure for each of the 300 persistent webs comprised of the 100 webs at the three levels of connectance.

We compared the structure of food webs among those primarily assembled by speciation, primarily assembled by invasion, and 19 well-known empirical food webs^57^ using 10 measures of network structure. One property is simply the number of species within the food web (*S*). Two other properties are standard measures of food-web trophic interaction richness^45^: links per species (*L*/*S*) also referred to as link density; and directed connectance (*C* = *L*/*S*^2^) which equals the proportion of all possible trophic links that are actually realized. Five more properties indicate the fraction of the following types of species in a food web: top (%*T*, species that have resource species but lack any consumer species^102^), intermediate (%*I*, species that have both resource and consumer species^102^), basal species (%*B*, species that have consumer species but lack resources species e.g., plants^102^); cannibals (*%C*, species that eat themselves)^43^; and omnivores (%*Omn*, species that eat species at different trophic levels)^43^. Trophic level is calculated as short weighted trophic level (*SWTL*), a measure of trophic level based on mere presence of links that accurately estimates trophic level based on quantitatively weighted links^103^. Two additional properties are the standard deviation of mean generality (*GenSD*) and vulnerability (*VulSD*) among species which quantify the variabilities of species’ normalized predator and prey counts respectively^43,104^.

We calculated 17 different measures of niche similarity between each introduced species and native species including similarity measured in terms of the number and fraction of shared consumer species (e.g., predators) and shared resource species (e.g., prey) between all native and introduced species and between all native and introduced species with which an introduced species shared one link. None of the 16 measures explained more variability of our results than “maximum similarity” measured as the similarity between the introduced species and the species in the web with which it shared the highest fraction of both predators and prey^43^. We classified each invasion sequence as invasion-dominated or speciation-dominated using the mean maximum similarity across all introduction events. We base this classification on the idea that new species generated by sympatric and parapatric speciation would be relatively similar to species already in the web while new species immigrating from the regional species pool would, on average, introduce species more trophically distinct and therefore less trophically similar^15^. Our use of mean maximum similarity recognizes that there are likely to be speciation and invasion events inconsistent with this pattern. Therefore, we are most interested in trends along the gradient from invasion-dominated to speciation-dominated webs labelled in terms of mean maximum similarity. We focus on several key questions related to differences in assembly dynamics across this gradient. First, is establishment success, which is defined as persistence to *t* = 10,000, different between speciation-dominated and invasion-dominated webs? Second, is turnover (proportion of extinctions relative to establishments) affected by mean maximum similarity? We were also interested in whether the final structure of the webs is different in webs dominated by invasion versus speciation. To answer this question, we focused on final species richness, final connectance, and final short-weighted trophic level. Differences between invasion and speciation dominated webs could be due to a number of differences in properties of the introduced species. In particular, we were interested in the relationship between average niche model parameters including *n*_*i*_*, c*_*i*_, and *r*_*i*_ and mean maximum similarity within the web. We also determined the relationship between mean maximum similarity and average invader vulnerability and generality. Finally, we also examined these food web properties to determine whether food web properties of webs dominated by invasion versus speciation differed from structural properties of 19 empirical food webs.

## Supplementary information

Supplementary Figure S1
