# Supplementary material for: Simulated evolution assembles more realistic food webs with more functionally similar species than invasion
Source: Sci Rep. 2019 Dec 3;9:18242. doi: 10.1038/s41598-019-54443-0 (PMC6890687; doi:10.1038/s41598-019-54443-0)
Supplement: Supplementary file 1 — Supplementary Figure S1 [file 41598_2019_54443_MOESM1_ESM.pdf]

Supplementary information for:

Simulated evolution assembles more realistic food webs with more functionally similar species than invasion

Tamara N. Romanuk[1,2], Amrei Binzer[3], Nicolas Loeuille[4],

W. Mather A. Carscallen[1], Neo D. Martinez[2,4*]

1 Department of Biology, Dalhousie University, Halifax, Canada

2 Pacific Informatics and Computational Ecology Lab, Berkeley, CA USA

3 Department of Physics, Chemistry and Biology, Linköping University, Linköping, Sweden

3 Institute of Ecology and Environmental Sciences, Université Pierre et Marie Curie, Paris, France

4 Department of Ecology and Evolutionary Biology, University of Arizona, Tucson, AZ USA

*contact: neo@peacelab.net

Supplementary Figure S1

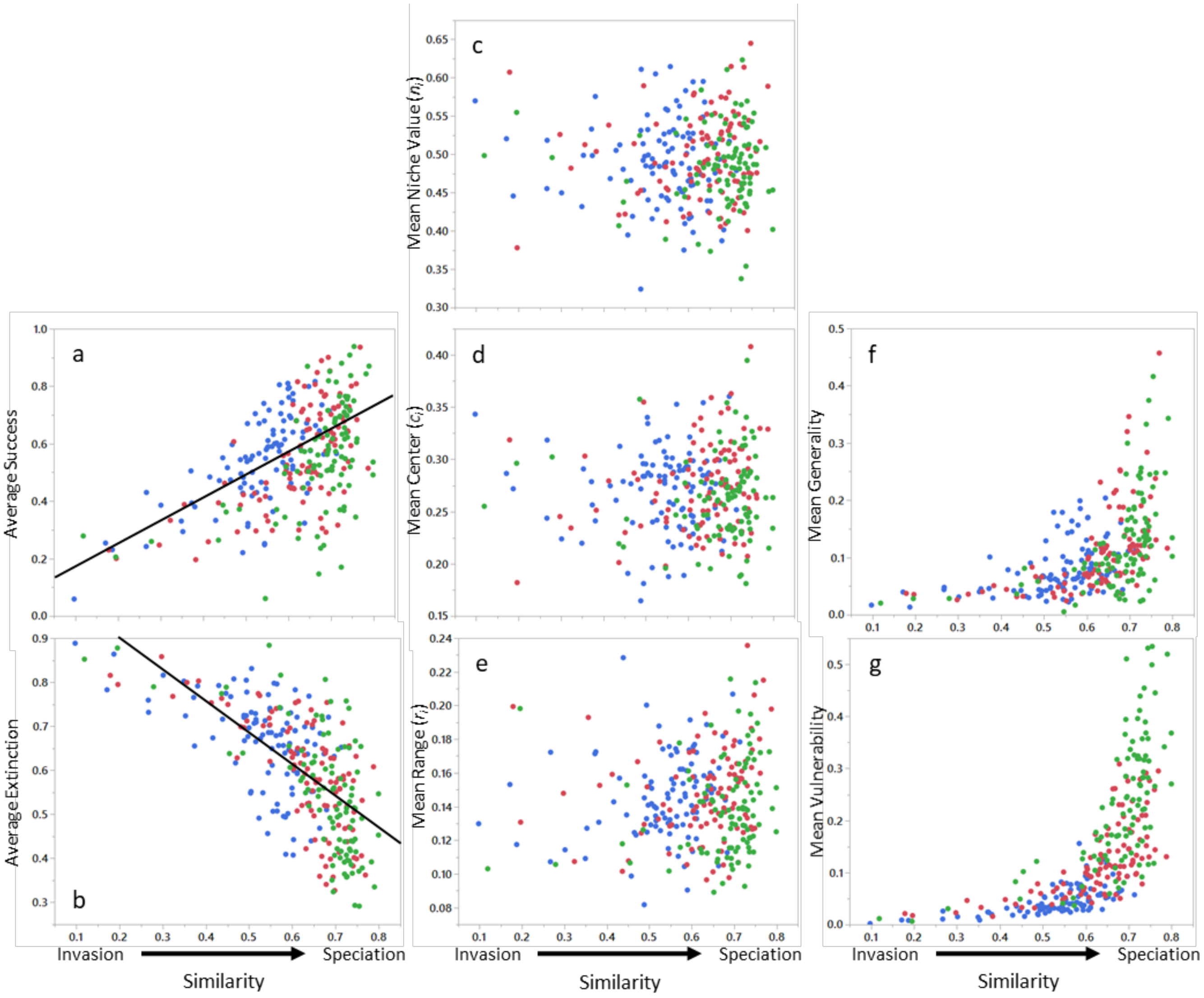

Figure S1. Revision of Figure 4 in the main text describing relationships between niche overlap and properties of introduced species color coded for data derived from low (blue), medium (gray) and high (green) connectance values.
